# Extracts from Plant Foods: Biological Characterization and Application

**DOI:** 10.3390/foods14244317

**Published:** 2025-12-15

**Authors:** Chengying Zhao, Jinkai Zheng

**Affiliations:** 1 Institute of Food Science and Technology, Chinese Academy of Agricultural Sciences, Beijing 100193, China; zhaochengying@caas.cn; 2 College of Food Science and Engineering, Qingdao Agricultural University, Qingdao 266109, China

Numerous plants with abundant diversity worldwide are rich in nutrients and functional components, including dietary fibers, polyphenols, flavonoids, alkaloids, etc. [1,2]. Many of them have been proven to possess special potential to maintain human health and exert various health-promoting effects, such as antioxidant, antibiotic, anti-inflammation, gastrointestinal protection, metabolism regulation, and cardiovascular protection [3]. By integrating their relatively high safety and renewability, the exploration and utilization of functional components derived from plant foods have attracted significant attention in the food industry [4]. However, several concerns must be addressed urgently. (1) Resource mining: Novel food resources from plant sources must be explored in order to address food resource shortages worldwide; meanwhile, the components in different plant samples vary dramatically depending on the variety, region, part (e.g., roots, stems, leaves, fruits, and seeds), and growth stage. Therefore, a comprehensive analysis of the components in diverse plant food resources should be carried out. (2) Separation and chemical structure analysis: Efficient target extraction techniques for special components and comprehensive utilization techniques for the simultaneous extraction of multiple components from plant foods must be developed, as well as techniques for structural identification and quantitative analysis. (3) Biological characterization: The health-promoting effects and mechanisms of plant-food-derived components should be systematically investigated, including the structure–activity relationship, in vivo metabolism, interactions among the gut microbiota, and targets.

This volume contains a collection of manuscripts on the extracts from plant foods. They demonstrated their differences among the raw materials with distinct varieties and growth periods. In addition, the effects of processing on the chemical structures, physicochemical properties, and health-promoting effects of plant extracts were also investigated. In detail, Cai et al. investigated the carotenoids and phenolics of the corn grain and corn husk of 15 yellow corn cultivars and 8 waxy corn cultivars, as well as their antioxidant activities. Four main carotenoid compounds including lutein, zeaxanthin, β-cryptoxanthin, and β-carotene were detected. Notably, they could only be detected in the yellow corn and black waxy corn, which could not be detected in other waxy corn. Lutein was the most abundant (494.5–2870.8 μg/g dw), followed by zeaxanthin (63.0–360.2 μg/g dw), β-cryptoxanthin, and β-carotene. The total content of polyphenols (TPC) and flavonoids (TFC) in the husk were all higher than those of the grain in almost all the corn cultivars except two yellow corn cultivars. The highest TPC was found in the husk of waxy corn. Sbii et al. explored the characteristic profiles, thermal properties, and oxidative stability of cold-pressed oils in the seed of moringa, milk thistle, and jujube. All the extracted oils exhibited high nutritional values, with significant differences among them. The oil of jujube seed showed the highest quality with an acidity equal to 0.8. At the same time, the content of polyphenols in jujube seed oil was highest, which exhibited high antioxidant activity compared with the other two seed oils. Thermal property assessment demonstrated that seed oils of milk thistle, jujube, and moringa were completely liquefied at 10, 15, and 30 °C, respectively. In addition, jujube seed oil showed the highest stability against thermal oxidation during storage for 60 days at 60 °C. Assefa et al. reported the agro-morphological traits and biochemical properties in glucosinolates (GSLs) of 355 leaf mustard. They showed significant variations in most of the traits. Sinigrin was the most abundant GSL followed by gluconapin and gluconasturtiin. Relationship analysis between agro-morphological traits and GSLs demonstrated that leaf length exhibited a negative correlation with the content of sinigrin and glucoiberin. The content of gluconapin, glucobrassicanapin, and glucobrassicin showed a positive correlation with the width of the petiole and midrib, while a negative correlation was found in the length of the petiole with the content of sinigrin, glucobrassicanapin, glucoiberin, glucobrassicin, and the total GSLs. Natthee et al. showed the potential of meal containing an anthocyanin-enriched functional ingredient at doses of 2 and 4 g per day for 5 days to alleviate anxiety, depression, and stress. The results demonstrated that the novel functional ingredient can decrease oxidative stress and inflammation by changing 8-OHdG and IL-6 levels, and thus improve anxiety, depression, and stress perception.

For the effects of processing on food quality and nutrient availability, Narra et al. investigated changes in the organoleptic characteristics, nutraceuticals, and antioxidant activity of tomato fruits during different thermal processing: tomato sauce (80 °C for 30 min), blanching treatment (100 °C for 10 s), and the superheated steam method (SHS; 100 °C for 7 min). This showed that although SHS negatively modified the color of the product, it could increase the antioxidant activity compared with fresh tomatoes, although the contents of lycopene and ascorbic acid were similar to that of fresh tomatoes. At the same time, extract from SHS exhibited similar antioxidant effects to that from fresh tomato in human endothelial cells. Rabiej-Kozioł and Szydłowska-Czerniak studied the effects of hydrolysis and lyophilization on the antioxidant activities of methanolic extracts from rapeseed meal. This showed that both alkaline and acid hydrolysis could significantly enhance the total phenolic content (TPC) and antioxidant activities compared to the control without hydrolysis treatment. Notably, the rapeseed meal extract after acid hydrolysis exhibited higher TPC and ferric reducing antioxidant power than alkaline hydrolysis. Lyophilization could further improve these effects. Moreover, the addition of acid-hydrolyzed and lyophilized rapeseed meal extract (HLRME) could improve the oxidation stability of refined rapeseed oil by decreasing the peroxide values, anisidine values, TOTOX and INTOX indexes, conjugated dienes, and total polar material. He et al. explored the interactions between soy protein isolate (SPI) and three folates, including folic acid (FA), L-5-methyltetrahydrofolate (MTFA), and calcium 5-methyltetrahydrofolate (CMTFA). This showed that folates could spontaneously bind to SPI by decreasing the Gibbs free energy and association constant. The thermodynamic parameters and molecular docking study revealed that the planar pteridine ring of FA and the conjugated double bonds could promote hydrophobic interactions, whereas the reduced ring structure and additional polar groups of MTFA enhanced the hydrogen bonding. The binding of SPI and folates showed the potential to stabilize the conformation to improve both the physical and thermal stability of the protein. Notably, compared with free FA, the combination of FA and SPI could increase the stability of FA. MTFA did not exhibit a comparable effect. This may be attributed to the distinct degradation pathways of FA and MTFA. This study provided both theoretical and experimental insights into the development of folate-loaded delivery systems based on SPI. Alvarado-López et al. investigated changes in the stability, bioactives, and antioxidant activity during cold storage (5 °C, 24 days) of a healthy Brazil nut beverage. Extracts of green *Opuntia stricta* var. *dillenii* pulp (ODP) were added to a standardized beverage at 0.5 and 1 g/100 g beverage. Most of the bioactive compounds from the green ODP extract added to the beverages showed good retention and remained stable throughout the 24 days of storage at 7 °C. The encapsulation efficiencies of betalains and phenolic compounds ranged from 98.3% to 92.4% and 93.7% and 81.2%, respectively. The resu lts showed the potential of Brazil nut beverage to be a healthy and efficient food emulsion system for the encapsulation of ODP extract.

In future studies, systematic investigations of the components, their health-promoting effects, and safety from different plant resources should be carried out. The exploration of novel plant resources used for food merits special attention. The variations and interactions of the plant components during processing should be studied. Based on that, novel green and efficient separation techniques should be considered for future development.

## Data Availability

Not applicable.

## References

[1] Pinheiro D.F., Maciel G.M., Lima N.P., Lima N.P., Ribeiro I.S., Haminiuk C.W.I. (2024). Impact of fruit consumption on gut microbiota: Benefits, contaminants, and implications for human health. Trends Food Sci. Technol..

[2] Cui J.F., Lian Y.H., Zhao C.Y., Du H.J., Han Y.H., Gao W., Xiao H., Zheng J.K. (2019). Dietary fibers from fruits and vegetables and their health benefits via modulation of gut microbiota. Compr. Rev. Food Sci. Food Saf..

[3] Li D., Zhang T., Lu J., Peng C., Lin L. (2021). Natural constituents from food sources as therapeutic agents for obesity and metabolic diseases targeting adipose tissue inflammation. Crit. Rev. Food Sci. Nutr..

[4] Rifna E.J., Misra N.N., Dwivedi M. (2023). Recent advances in extraction technologies for recovery of bioactive compounds derived from fruit and vegetable waste peels: A review. Crit. Rev. Food Sci. Nutr..

